# Correction to: Children and Wild Foods in the Context of Deforestation in Rural Malawi

**DOI:** 10.1007/s10745-018-9976-z

**Published:** 2018-03-01

**Authors:** H. Maseko, Charlie M. Shackleton, J. Nagoli, D. Pullanikkatil

**Affiliations:** 1 Department of Environmental Science, Rhodes University, Grahamstown, 6140 South Africa; 2 WorldFish – Malawi, P. O Box 229, Zomba, Malawi

**Correction to: Hum Ecol (2017) 45:795–807**

10.1007/s10745-017-9956-8

Information on research funding presented in the paper by Maseko *et al.*, “Children and wild foods in the context of deforestation in rural Malawi” is incorrect. The correct funding credits should read:

**Funding** We acknowledge financial contributions for the fieldwork and bursary (to HM) from the ASSETS project (NE-J002267–1) funded under the ESPA programme of DFID, ESRC and NERC, as well as the National Research Foundation (South Africa).

The article Children and Wild Foods in the Context of Deforestation in Rural Malawi, written by H. Maseko, Charlie M. Shackleton, J. Nagoli and D. Pullanikkatil, was originally published Online First without open access. After publication in volume 45, issue 6, page 795–807 the author decided to opt for Open Choice and to make the article an open access publication. Therefore, the copyright of the article has been changed to © The Author(s) 2017 and the article is forthwith distributed under the terms of the Creative Commons Attribution 4.0 International License (http://creativecommons.org/licenses/by/4.0/), which permits use, duplication, adaptation, distribution and reproduction in any medium or format, as long as you give appropriate credit to the original author(s) and the source, provide a link to the Creative Commons license, and indicate if changes were made.

